# 

**Published:** 1869-08

**Authors:** 


### RESIGNATION—APPOINTMENT.

Prof. J. A. Watling, who so efficiently and acceptably filled the chair of Clinical Dentistry in the Ohio College of Dental Surgery during the last year, has, by various circumstances, been compelled to resign that position. This announcement will cause regret to every friend of the institution, who knows Prof. W.'s ability. But we are gratified to state that the chair has been filled with so good and acceptable a member of the profession as Dr. A. M. Moor, of Lafayette, Ind. Dr. Moor is favorably known to the whole profession of this county, and especially in the West.

We consider the College very fortunate in securing Dr. M. as one of its teachers. T.

The Dental Register

OF THE WEST,

*Issued Monthly, at $3 a Year in Advance.*

DEVOTED TO THE INTERESTS OF THE DENTAL PROFESSION.

EDITED BY J. TAFT AND G. WATT.

The volume commences in January and closes in December.

The Register being issued monthly is always fresh, therefore indispensable to the practitioner who desires to keep posted up with the new inventions and improvements which are daily developed. Neither expense nor effort will be spared to give value and variety to its contents. Articles will be illustrated with well executed engravings whenever they will add to the interest or assist in explanation.

Its extensive circulation and frequency of issue makes it one of the BEST DENTAL ADVERTISING MEDIUMS in the United States.

RATES OF ADVERTISING.

One page, 1 year, $50. Half page, 1 year, $30. Quarter page, or card, 1 year $20.

All advertising bills payable in advance, or at furthest after the issue of the first number containing the advertisement. All transient advertisements to be paid for in advance.

☞ CONTRIBUTIONS SOLICITED.

Address, J. TAFT, or "DENTAL REGISTER," Cincinnati Ohio.

Business Communications to

**J. TAFT, Publisher,**

No. 117 West Fourth St.

